# Diversity of Plastid Types and Their Interconversions

**DOI:** 10.3389/fpls.2021.692024

**Published:** 2021-06-17

**Authors:** Heebak Choi, Taegyu Yi, Sun-Hwa Ha

**Affiliations:** Department of Genetics and Biotechnology, Graduate School of Biotechnology, College of Life Sciences, Kyung Hee University, Yongin, South Korea

**Keywords:** chloroplast, chromoplast, de-greening, etioplast, greening, leucoplast, non-greening

## Abstract

Plastids are pivotal subcellular organelles that have evolved to perform specialized functions in plant cells, including photosynthesis and the production and storage of metabolites. They come in a variety of forms with different characteristics, enabling them to function in a diverse array of organ/tissue/cell-specific developmental processes and with a variety of environmental signals. Here, we have comprehensively reviewed the distinctive roles of plastids and their transition statuses, according to their features. Furthermore, the most recent understanding of their regulatory mechanisms is highlighted at both transcriptional and post-translational levels, with a focus on the greening and non-greening phenotypes.

## Introduction

Plastids first developed during an endosymbiotic event between photosynthetic prokaryotes and the eukaryotic ancestors of algae. During the subsequent co-evolution of the engulfed plastid and eukaryote cells there were extreme changes in the functions of the plastids, including the development of regulatory networks (Keeling, 2013; Ševćíková et al., 2015). Although plastids are common subcellular organelles in plants, previous research has been a bias toward the photosynthetic plastids called chloroplasts or carotenoid enriched plastids called chromoplasts (Cruz et al., 2018; Pinard and Mizrachi, 2018). Moreover, recent studies on the regulatory pathways of plastids have mainly focused on light switching and hormonal treatments (López-Juez, 2007; Larkin and Ruckle, 2008; Larkin, 2014; Al-Babili and Bouwmeester, 2015; Liu et al., 2017).

In this review, the distinctive features for each plastid type are briefly described to help understand the general properties of plastids. A point of interest is that new plastids cannot be generated or born, and are duplicated or transited from other plastids. This indicates that the interconversion of plastids is important, and this is consequently addressed in this review. However, as plastid interconversions are tissue- and species-specific events, representative research was carefully selected and summarized.

Although there are many cases of plastid interconversions, the molecular mechanisms in the signaling networks of chloroplasts and chromoplasts were the focus of this review. During chloroplast development, different wavelengths of light synergistically trigger transcriptional regulators by releasing the post-translational inhibition of E3 ligase complexes. This leads to an increase in transcriptional regulators which induce photomorphogenic enzymes and chloroplast development. Chromoplasts are differentiated by the induction of carotenoid biosynthesis genes through the induction of transcriptional regulators. In this review, the chloroplast and chromoplast molecular networks are described with newly discovered regulators at both transcriptional and post-translational levels. Together with general examples of plastid interconversion, the overview of gene-specific molecular networks and their participating genes will strongly support work toward genetic improvement of multiple traits which related to plastid interconversion.

## Plastid Types and Roles

**FIGURE 1:**
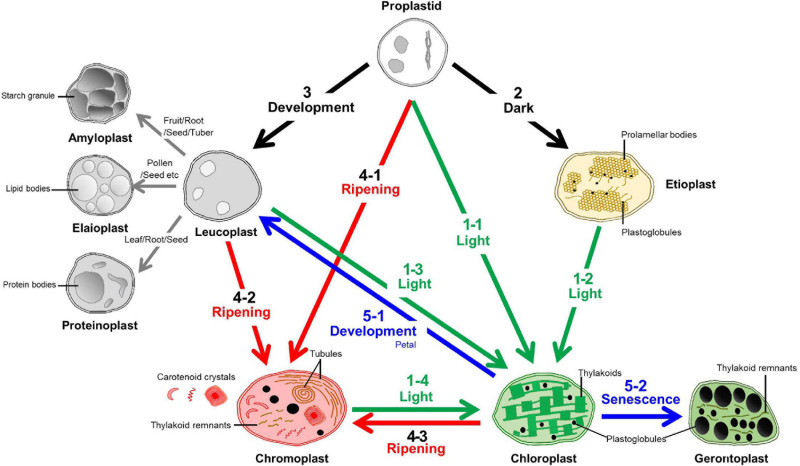
Transition pathways among various plastids. The characteristics and plastid interconversion pathways of the plastids were classified according the color and number of the arrow. The transition to a chloroplast is called “Greening” and identified with the number “1”. This is mainly triggered by light signals from proplastids, etioplasts, leucoplasts, and chromoplasts. Etioplasts can develop from proplastids in dark conditions and this identified by the number “2”. The number “3” indicates leucoplast development that is triggered by diverse development processes to generate starch, lipid, and protein enriched sub-types called amyloplasts, elaioplasts, and proteinoplasts, respectively. Mainly during the ripening stage, diverse types of the carotenoid crystals were generated within the plastids called chromoplasts from the proplastids, leucoplasts, and chloroplasts and this is identified with the number “4”. Together with etioplast and leucoplast development (2,3), chromoplast development (4) was identified as a “Non-greening” plastid transition. The loss of green color from the chloroplasts is called “De-greening” and identified with the number “5”, and these chloroplasts are then transited into leucoplast or gerontoplast by developmental regulation or during senescence, respectively.

Plastids can be divided into several types based on their color, morphology, and ultrastructure (Whatley, 1978; Møller, 2006; Wise, 2007). The characteristics of each plastid type are tightly related to their specific roles (Figure 1). Undifferentiated plastids are called “proplastids” and are mainly found in meristematic and reproductive tissues, and they are identified as being small and having clear ultrastructures. They can be differentiated into “leucoplasts” in white, “chloroplasts” in green, and “chromoplasts” in either yellow, orange or red. Intermediate forms of chloroplasts are called “etioplasts” and senescent forms of chloroplasts are called “gerontoplasts”. Leucoplasts are categorized by their lack of color, but can be further separated according to their biochemical characteristics based on their contents, such as starch enriched “amyloplasts”, protein enriched “proteinoplasts”, and lipid enriched “elaioplasts” (Lopez-Juez and Pyke, 2004; Jarvis and López-Juez, 2013).

### Proplastids

Proplastids are undifferentiated plastids that maintain a minimal plastid structure. So that, their organelle transmission can take place between generations. They are colorless and tiny in size when compared to the other types of plastid with no significant morphological characteristics (Jarvis and López-Juez, 2013; Liebers et al., 2017). They are mostly found in meristematic and egg cells of plants and sometimes during pollen formation in specific species such as *Pelagonium* and barley (*Hordeum vulgare*) (Hagemann, 2004; Sakamoto et al., 2008; Gajecka et al., 2020). Also, the nodule proplastids in root tissues have been reported to play a vital role in the biochemistry of nitrogen fixation in the legume family (Boland and Schubert, 1983; Ferguson, 1998; Greco et al., 2012).

### Chloroplasts

Chloroplasts are one of the most well-studied types of plastid and are found in all photosynthetic organisms (Waters and Langdale, 2009; Rottet et al., 2015). They can turn light energy into chemical energy via photosynthetic protein complexes. In chloroplasts, multiple stacks of disk-like single lipid layers called thylakoids form grana and these create large lipid surface layers which anchor the photosynthetic protein complexes. The edges of the disk-like thylakoids also form unique hydrophobic pocket structures called plastoglobules, which help to enlarge the internal area of the lipid bilayer (Rottet et al., 2015). Plastoglobules are identified as sites of carotenoid breakdown for apocarotenoid production (Rottet et al., 2016) and for non-endogenous carotenoid accumulation (Mortimer et al., 2017).

The green color of chloroplasts is due to chlorophyll which is core component for photosynthesis, but the chloroplasts also have an abundance of multiple hydrophobic terpenes, such as lutein, β-carotene, violaxanthin, and neoxanthin, which also help to support photosynthesis (Ruiz-Sola and Rodríguez-Concepción, 2012). Not only converting the UV-blue range of light to the electro energy for photosynthesis (Dall’Osto et al., 2014), the carotenoid in chloroplast also play a major role in photoprotection by modulating the non-radiative dissipation of excess excitation energy (Niyogi, 2000; Dall’Osto et al., 2005). Specially, hydroxylated carotenoids referred to as xanthophylls support photoprotection by mediating direct quenching of chlorophyll (Chl) triplets (^3^Chl^∗^) or by scavenging the reactive oxygen species (ROS) generated during photosynthesis (Niyogi et al., 1997; Havaux and Niyogi, 1999; Dall’Osto et al., 2007). Consequently, the balance between photosynthesis, photoprotection, and ROS scavenging is an important function in chloroplasts. Starch granules, protein bodies, and lipid bodies are often formed in chloroplasts for temporal storage and to help meet the demands of developmental and environmental cues.

### Etioplasts

Etioplasts are specialized intermediate plastid types that are mostly found in dark grown seedlings. In natural conditions, they are easily found in seedlings that grow under the soil. They are the transient state of development for chloroplasts and are also considered as a status of austerity because they stop the development of photosynthetic chemicals and structures which are unnecessary in the dark. Within etioplasts, in general, single well-arranged paracrystalline prolamellar body and tubular prothylakoids are formed, and these are interspersed with numerous small plastoglobule with high amounts of carotenoids; mainly lutein and violaxanthin which help to increase the transition to chloroplasts (Park et al., 2002; Rodríguez-Villalón et al., 2009; Solymosi and Schoefs, 2010; Pipitone et al., 2021).

### Leucoplasts and Derivatives

Leucoplast are characterized by their white structures (Carde, 1984). They are often found in non-photosynthetic tissues that have storage functions. However, advances in microscopy technology and an increase in detection strategies have made it possible to classify leucoplast in more detail. Except for undeveloped proplastids, three types of white plastids, amyloplasts, proteinoplasts, and elaioplasts, are further characterized as sub-types of leucoplasts (Howitt and Pogson, 2006; Sadali et al., 2019).

### Amyloplasts

Amyloplasts are characterized by starch granules that store high density starch. During the formation of amyloplast membranes, various lipids such as free fatty acids, lysophospholipids, lysophosphatidylcholine, and lysophosphatidylethanolamine are also included in the starch granules (Gayral et al., 2019). Amyloplasts are commonly found in sink tissues including seeds, fruits, tubers, and roots for carbon storage, but they are also often found at low frequencies, in various tissues including leaves, stems, and roots for temporal storage (Jarvis and López-Juez, 2013). It is interesting that the amyloplasts is not stable in some cases, for example in Arabidopsis leaves, the accumulation and the loss of starch are highly dynamic, following a daily cycle due to photosynthetic activity or its absence (Fernandez et al., 2017). Unlike other types of plastid, amyloplasts often coexist with different types of plastid in the same cell. However, in the tissues of species, such as the winter squash, peach palm fruit, and sweet potato tuber (Jeffery et al., 2012; Hempel et al., 2014; Zhang et al., 2014), combinatory types of plastid called amylochromoplasts were observed which stored starch granules with carotenoid crystals in the same plastid. Starch granules are also found inside different types of plastids such as chloroplasts. As well as their storage functions, the amyloplast from Arabidopsis roots were reported to contribute to gravitropism signaling (Chen et al., 1999; Nakamura et al., 2019).

### Elaioplast

Elaioplasts are characterized by ultrastructures filled with hydrophobic contents such as lipids and terpenoids. They are specialized for biosynthesis and the storage of lipids, but also have diverse functions in specific tissues. In citrus fruits, elaioplasts are exported into secretory pockets and they can have large impacts on aroma and taste (Zhu et al., 2018). In pollen, exine formation was found to be highly dependent on elaioplasts (Quilichini et al., 2014).

### Proteinoplasts

In specific cases, protein bodies can be found in plastid structures, generally in the cytosolic area, and these are called proteinoplasts (or proteoplast, aleuroplast, aleuronaplast; Dashek and Miglani, 2017). Proteinoplasts are generally found in many different types of cell at several different stages of plastid development (Thomson and Whatley, 1980). Due to the location and contents of proteinoplasts, they are thought to have a role in protein storage. Furthermore, the proteinoplasts of tobacco root showed strong oxidase activity which may convey a specific function (Vigil and Ruddat, 1985).

### Chromoplasts

Chromoplasts have colorful characteristics as they accumulate large amounts of carotenoids and their specific colors are determined by specific types of carotenoids. During chromoplast development, the concentrated carotenoids which form globular, round, coiled shaped carotenoid crystals at the mature stage are produced and stored in hydrophobic structures called plastoglobules (Schweiggert et al., 2011). The plastoglobules are lipoprotein particles attached to thylakoids through a half-lipid bilayer and function in both lipid biosynthesis, storage and cleavage (Austin et al., 2006; Rottet et al., 2016). These colored plastids with highly developed plastoglobules are used to attract pollinators and seed disseminators in reproductive tissues or for the storage of carotenoids and hydrophobic metabolites (Rottet et al., 2015).

### Gerontoplasts

Gerontoplasts are chloroplast-derived plastids adjusted for recycling of plastid which are mainly found during senescence processes or under stress condition (Biswal et al., 2012). As the chloroplast possess up to 80% of the leaf nitrogen pool, degradation of chloroplasts and the recycling of their nutrients is important for plant survival (Makino and Osmond, 1991). And the degradation of chloroplast proteins has been reported for three different pathways, autophagy, Senescence Associated Vacuoles (SAV) and Chloroplast Vesiculation (CV) (Ishida et al., 2008; Xie et al., 2015). When the senescence process starts, plastids undergo serial changes in their ultrastructures. It is difficult to define the characteristics of gerontoplasts at the beginning of senescence, but there are a few specific characteristics that have been identified (Biswal et al., 2012). First, gerontoplasts do not contain starch granules, probably because they are unable to continue photosynthesis which replenish the starch daily. Second, their thylakoid structures and chlorophyll have also been degraded. Third, the size of their plastoglobules is enlarged and their numbers increased, probably due to the accumulation of lipophilic substances from degraded lipid structures and hydrophobic contents.

### Specialized Types: Desiccoplasts, Phenyloplasts, and Xyloplasts

Desiccoplasts are plastids that can be interconverted between chloroplasts and proplastids in desiccation tolerant plants (Solymosi et al., 2013). Phenyloplasts are phenol enriched colorful plastids identified as a new plastid type when compared to chromoplasts because of their different storage contents and the homeostatic roles of phenols (Brillouet et al., 2014). Xyloplasts are specialized plastids in secondary vascular tissues that are dedicated to the synthesis of precursors for monolignol production, derived from either proplastids, or more likely, amyloplasts (Pinard and Mizrachi, 2018).

## Plastid Transitions

### Development of Chloroplast: Greening Phenotype

#### From Proplastids

Transitions of proplastids to chloroplasts mainly occur in the shoot apical meristem and during embryogenesis (Table 1). According to Arabidopsis research, the process of differentiation starts with the shoot apical meristem of the young leaf and continues into leaf development. The differentiation process occurs in the upper layer and central subtending cell layers, and is not affected by the intensity of light, but a light period of 5–10 h is required (Yadav et al., 2019). By observing embryonic development in Arabidopsis, chloroplast-containing cells were identified at the globular stage of embryogenesis, indicating the development of chloroplasts from undifferentiated proplastids (Tejos et al., 2010). In *in vitro* experiments, dark grown calluses only had proplastids while those grown in the light had short thylakoids and chloroplasts containing an immature membrane structure (Ladygin et al., 2008).

**TABLE 1:** Plastid transitions according to plant phenotypes in the view of greening status.

**Plastid transition**				**Scientific name (common name)**	**Organ/tissue/cell**	**References**
**Phenotype**	**Pathway**	**From**	**To**			
Greening	1-1	Proplastid	Chloroplast	*Arabidopsis thaliana* (Arabidopsis)	Shoot apical meristem	Yadav et al., 2019
				*Arabidopsis thaliana* (Arabidopsis)	Embryo	Tejos et al., 2010
				*Stevia rebaudiana* (Stevia, *in vitro)*	Callus	Ladygin et al., 2008
	1-2	Etioplast	Chloroplast	*Arabidopsis thaliana* (Arabidopsis)	Leaf	Pipitone et al., 2021
				*Brassica oleracea* (Cabbage)	Leaf	Solymosi et al., 2004
				*Cucumis sativus* (Cucumber)	Cotyledon	Sobieszczuk-Nowicka et al., 2007
				*Lactuca sativa* (Lettuce)	Leaf	Kanamoto et al., 2006
				*Nicotiana tabacum* (Tobacco)	Leaf	Armarego-Marriott et al., 2019
	1-3	Leucoplast	Chloroplast	*Arum italicum* (Italian Arum)	Fruit	Bonora et al., 2000
				*Solanum tuverosum* (Potato)	Tuber	Tanios et al., 2018
				*Picea abies* (Norway Spruce)	Needle leaf	Senser et al., 1975
	1-4	Chromoplast	Chloroplast	*Citrus sinensis* (Citrus fruit)	Fruit	Mayfield and Huff, 1986
				*Cucumis sativus* (Cucumber)	Fruit pericarp	Prebeg et al., 2008
				*Cucumis maxima* (Cucumber)	Subepidermal cell of fruit	Rodriguez-Concepcion and Stange, 2013
				*Cucurbita pepo* (Pumpkin)	Subepidermal cell of fruit	Devidé and Ljubešiæ, 1974
				*Daucus carota* (Carrot)	Root	Rodriguez-Concepcion and Stange, 2013
Non-greening	2	Proplastid	Etioplast	*Arabidopsis thaliana* (Arabidopsis)	Leaf	Bykowski et al., 2020
				*Phaseolus vulgaris* (Bean)	Hypocotyl	Kakuszi et al., 2017
	3-1	Proplastid	Amyloplast	*Arabidopsis thaliana* (Arabidopsis)	Root	Kiss et al., 1989
				*Arum italicum* (Italian Arum)	Fruit	Bonora et al., 2000
				*Linum usitatissimum* (Flax)	Root	Kuznetsov and Hasenstein, 1996
				*Malus pumila* (Apple, *in vitro*)	Callus	Sagisaka, 2008
				*Musa acuminata* (Banana)	Fruit	Solis-Badillo et al., 2020
				*Oryza sativa* (Rice)	Endosperm	Matsushima et al., 2014
				*Pisum sativum* (Pea)	Root	Hodson and Mayer, 1987; Borchert et al., 1989
				*Solanum tuverosum* (Potato)	Stolon	Sagisaka, 2008
				*Solanum tuverosum* (Potato)	Tuber	Naeem et al., 1997
				*Zea mays* (Maize)	Endosperm	Wurtzel et al., 2012
	3-2	Proplastid	Elaioplast	*Althaea rosea*	Root, Hypocotyl	Kwiatkowska et al., 2011
				*Arabidopsis thaliana* (Arabidopsis)	Pollen	Kobayashi and Masuda, 2016
				*Brassica napus* (Rapeseed)	Seed	Howitt and Pogson, 2006
				*Centaurea cyanus* (Cornflower)	Secretory duct of stem	Pacek et al., 2012
				*Citrus sinensis* (Citrus fruit)	Outer peel of the fruit	Zhu et al., 2018
				*Haemanthus albiflos*	Leaf epidermis	Kwiatkowska et al., 2010
				*Helianthus annuus* (Sunflower)	Seed	Howitt and Pogson, 2006
				*Persea americana* (Avocado)	Mesocarp of the fruit	Scott et al., 1963
				*Vanilla planifolia*	Young leaf	Kwiatkowska et al., 2011
	3-3	Proplastid	Proteinoplast	*Helleborus corsicus* (Helleborus)	Leaf	Härtel and Thaler, 1953
				*Nicotiana tabacum* (Tobacco)	Root	Vigil and Ruddat, 1985
				*Vigna radiata* (Mung bean)	Leaf	Dashek and Miglani, 2017
				*Zea maize* (Maize)	Seed	Denic et al., 1971
	4-1	Proplastid	Chromoplast	*Carica papaya* (Papaya)	Fruit	Schweiggert et al., 2011
				*Citrullus lanatus* (Watermelon)	Fruit	Fang et al., 2020
				*Daucus carota* (Carrot, *in vitro)*	Callus	Oleszkiewicz et al., 2018
	4-2	Leucoplast	Chromoplast	*Brassica oleracea* (Cauliflower)	Flower curd in mutant	Paolillo et al., 2004
				*Daucus carota* (Carrot)	Root	Kim et al., 2010
				*Oryza sativa* (Rice)	Transgenic	Bai et al., 2016
				*Zea mays* (Maize)	Endosperm	Wurtzel et al., 2012
	4-3	Chloroplast	Chromoplast	*Arum italicum* (Italian Arum)	Fruit	Bonora et al., 2000
				*Capsicum frutescens* (Pepper)	Fruit	Jeong et al., 2020
				*Lilium longiflorum* (Lily)	Flower	Juneau et al., 2002
				*Solanum lycopersicum* (Tomato)	Fruit	Wang et al., 2020
De-greening	5-1	Chloroplast	Leucoplast	*Arabidopsis thaliana* (Arabidopsis)	Flower petal	Pyke and Page, 1998
	5-2	Chloroplast	Gerontoplast	*Arabidopsis thaliana* (Arabidopsis)	Leaf	Evans et al., 2010
				*Jatropha curcas* (Jatropha)	Seed inner integument	Shah et al., 2016

*Pathway numbers correspond to those in the schematic diagram in Figure 1.*

#### From Etioplasts

When etioplasts are exposed to light, protochlorophyllide, the chlorophyll precursor of prolamellar bodies, is immediately converted to chlorophyllide by light-dependent NADPH:Pchlide oxidoreductase. Following this, chlorophyllide is converted to chlorophyll through enzymatic processes (Fujii et al., 2019). It occurs mainly in plant leaf tissues and can be easily found in the plant world, for example, the inner leaves of white cabbage (*Brassica oleracea* “Capitata”), lettuce (*Lactuca sativa*) and cucumber cotyledons (*Cucumis sativus*) (Solymosi et al., 2004; Kanamoto et al., 2006; Sobieszczuk-Nowicka et al., 2007). De-etiolation studies using tobacco leaves reported that the physical structures of the etioplast prolamellar bodies changed almost immediately when exposed to light, and regularity and size reductions occurred (Armarego-Marriott et al., 2019). Recent work well described in Arabidopsis revealed the chloroplast biogenesis from etioplast into two distinct phases: the “Structure Establishment Phase” for disassembly of the prolamellar body, gradual formation of the thylakoid membrane and initial increase of galactolipids and photosynthesis-related proteins and the “Chloroplast Proliferation Phase” for cell expansion, a linear increase of prokaryotic and eukaryotic galactolipids, photosynthesis-related proteins and increased grana stacking (Pipitone et al., 2021).

#### From Leucoplast

Non-photosynthetic leucoplasts can be converted to photosynthetic chloroplasts. In the cortical parenchyma tissues of potato tubers, directly beneath the periderm, amyloplasts (starch enriched leucoplast) are converted to chloroplasts by the accumulation of chlorophylls under light sources (Tanios et al., 2018). In addition, the needle leaf of Norway spruce (*Picea abies*) also showed the plastid transitions according to growth and seasonal changes. In Norway spruce, amyloplasts for nutrient accumulation and chloroplasts for photosynthesis are generated at the seedling stage. Seasonally, amyloplasts appear mainly due to the accumulation of large amounts of starch in the autumn and winter and are converted back to typical chloroplasts in the spring and summer (Senser et al., 1975). For Italian arum (*Arum italicum*) fruits, greening proceeds after the fruits are formed. As the green color emerges, the amyloplasts are converted to chloroplasts with thylakoid membrane development and the plastoglobules increase in number and size (Bonora et al., 2000).

#### From Chromoplasts

Reversible changes from chromoplast to chloroplast are called regreening and can be found in citrus fruits (Mayfield and Huff, 1986), pumpkin (Devidé and Ljubešiæ, 1974), and cucumber fruits (Prebeg et al., 2008; Rodriguez-Concepcion and Stange, 2013). Cucumber thylakoids are decomposed as the fruit matures, and then the thylakoid is reconstituted due to regreening. The plastids of mature fruit and the regreened chloroplasts show morphological similarities, which means re-differentiation of the plastids. Reconstitution of the thylakoids begins with membrane-bound bodies, and surface expansion and fragmentation occur. Afterward, tubules and double-membrane sheets are formed. The plastoglobuli remain in the plastid even during reconstruction. This transition implies that several types of membrane structures are associated with the plastid envelope during chloroplast re-differentiation (Prebeg et al., 2008). Light is regarded as a key factor that greatly influences regreening. As a result of irradiating the citrus fruit Valencia orange with a blue LED light, the tissue was gradually greened, and after 4 weeks, the chlorophyll content was approximately twice as high as that of the unirradiated tissue (Ma et al., 2020). In addition, the roots of the carrots were also completely altered by exposure to the light, and converted from β-carotene-rich chromoplasts to lutein-containing chloroplasts (Rodriguez-Concepcion and Stange, 2013).

### Development of Proplastids Into Etioplasts: Non-greening Phenotype

When seeds are buried underground without light, but with sufficient environmental conditions for germination, the proplastids can develop into etioplasts while the plants etiolate. This transition is widely adopted by most plants and is an efficient strategy for seedlings in light-seeking circumstances. Until the photosynthetic tissues reach a light source, etioplasts develop with stacking prolamellar bodies and numerous small plastoglobuli (Rodríguez-Villalón et al., 2009). According to studies on Arabidopsis and soybeans, etioplast formation is influenced by etiolation time, and the efficient tubular-lamellar arrangement affects subsequent vegetative growth (Kakuszi et al., 2017; Bykowski et al., 2020). The key element to maintaining etioplasts is the completely dark environment. The loss of negative regulators of photomorphogenesis (DET1, COP1, and a combination of PIFs) inhibited etioplast differentiation in dark conditions, thereby suggesting that etioplasts develop in dark conditions via the negative regulation of photomorphogenesis (Wei et al., 1994; Sperling et al., 1998; Stephenson et al., 2009).

### Development of Proplastids Into Leucoplasts: Non-greening Phenotype

#### To Amyloplasts

The development of amyloplasts can be observed in most tissues with high-starch contents. Starch is often stored in the root tissues of plants, such as with Arabidopsis (Kiss et al., 1989), flax (Kuznetsov and Hasenstein, 1996), and pea (Hodson and Mayer, 1987; Borchert et al., 1989). Furthermore, the tubers and stolons of potatoes are representative organs that accumulate amyloplasts (Naeem et al., 1997; Sagisaka, 2008). In the case of fruits, starch mainly accumulates during the “maturation” period, such as with banana (Solis-Badillo et al., 2020) and Italian arum (Bonora et al., 2000). For apples, *in vitro* experiments showed that the formation of amyloplasts occurs in the callus and endosperm (Sagisaka, 2008). For rice and wheat, the accumulation of amyloplasts mainly found in the endosperm (Wurtzel et al., 2012). In amyloplasts, starch is produced in the matrix space (stroma) and forms starch grains, which exhibit different morphologies depending on the plant species and have been intensively studied in various staple crops. The diameter of starch grains in corn, rice and sorghum are about 10, 10–20, and 15–25 μm, respectively, while less than 10 μm in barley and wheat (Matsushima et al., 2014; Matsushima and Hisano, 2019).

#### To Elaioplasts

The elaioplasts have been largely reported in flowers and they can be found in their ovaries, ovary epidermis, and innermost tapetum cell of the anther wall (Chen et al., 1988; Ciampolini et al., 1993; Kwiatkowska et al., 2010, 2011). Elaioplasts were also reported in secretory ducts of the stem and leaf epidermis from *Centaurea cyanus* and *Haemanthus albiflos* (Kwiatkowska et al., 2010; Pacek et al., 2012), young leaves of *Vanilla planifolia*, roots, hypocotyls of *Althaea rosea* (Kwiatkowska et al., 2011), seeds of canola and sunflower (Howitt and Pogson, 2006; Lersten et al., 2006), mesocarp of the fruit in avocados (Scott et al., 1963), and green pericarps of citrus fruits (Zhu et al., 2018). The formation of elaioplasts has been reported to occur by a diverse range of mechanisms that vary by species. Observations of elaioplast differentiation in the tapetal cells of *Arabidopsis thaliana*, showed that the transition from proplastid to elaioplast occurred at “stage 9” with spot like structures that shapes like plastoglobuli (Suzuki et al., 2013).

#### To Proteinoplast

Proteinoplasts have been studies in detail from the roots of tobacco (Vigil and Ruddat, 1985). They are mainly distributed in the vacuolate and root cap cells of the root and accumulate in the slender tubules of the plastids. As cells divide, protein accumulation occurs, tubules expand, and protein bodies of dense spheroidal structures appear. Proteinoplasts were also observed in the leaves and seeds of *Helleborus corsicus* and *Zea maize*, respectively (Härtel and Thaler, 1953; Denic et al., 1971). In addition, a proteinoplast with a granular matrix containing a large amount protein was reported in the leaves of mung bean (Dashek and Miglani, 2017).

### Development of Chloroplast Into Chromoplasts: Non-greening Phenotype

#### From Proplastid

The transition from proplastid to chromoplast is often found during fruit maturation. Representative examples of the transition from proplastid to chromoplast can be found in watermelon (*Citrullus lanatus*), papaya (*Carica papaya*), and carrot calluses. In papaya during early white maturation, undifferentiated proplastids and globular plastids were dominant, but intermediate plastids such as the chloroplasts and amyloplasts were not found until chromoplasts developed. They were thus thought to have differentiated from the proplastids (Schweiggert et al., 2011). In watermelon, similar chromoplast development with an analysis of the pattern of each color step has been reported. When looking at the plastid differentiation patterns of watermelon, the accumulation of carotenoids and chromoplasts appeared according to the maturity of the fruit. In addition, many plastoglobuli were accumulated in the chromoplasts of yellow and orange watermelons when compared to white watermelons. The red watermelon chromoplasts formed an elongated or irregular structure, and the number of plastoglobuli further increased (Fang et al., 2020). In the carrot callus system, proplastids could be converted into chromoplasts during callus differentiation. In the case of pale-yellow carrot calluses, the carotenoid content was low while most of the plastids were proplastids. Conversely, for dark-orange carrot calluses, there were a large amount of chromoplasts, with high carotenoid contents, while the number of proplastids was significantly reduced (Oleszkiewicz et al., 2018). Meanwhile, the Arabidopsis callus contains proplastids, but the induced or stable overexpression of a phytoene synthase gene (PSY) showed the increased carotenoid contents with the chromoplast development (Maass et al., 2009; Rodríguez-Villalón et al., 2009).

#### From Leucoplasts

This transition occurs during the maturation of fruits, flowers, and roots (Sun et al., 2018). Relatively well-studied cases for this transition are carrots (*Daucus carota*) and orange cauliflower mutants (*Brassica oleracea* L. var. botrytis). In the case of carrot roots, the types of plastids present differed markedly depending on the color. The root of orange carrots was rich in chromoplasts with crystal-shaped structure due to carotene, whereas white carrots had fewer chromoplasts and no crystal-shaped structure. Instead, the white carrot roots showed amyloplasts filled with starch grains, and the total number of the chromoplasts and amyloplasts did not show any significant differences (Kim et al., 2010). In the case of orange cauliflower, the plastid of white wild-type tissues was characterized by leucoplasts, where orange-colored mutants have chromoplasts with accumulated β-carotene (Paolillo et al., 2004). Chromoplast transitions could also be found in the endosperms of rice and corn, which are mostly formed by amyloplasts. Although wild-type rice endosperms do not produce carotenoids, a combination of multiple carotenogenic genes such as *1-deoxy-D-xylulose 5-phosphate synthase* (*DXS*), *PSY*, *bacterial phytoene desaturase* (*CRTI*), and *ORANGE* (*OR*) could result in chromoplast development (Ye et al., 2000; Wurtzel et al., 2012; Bai et al., 2016; You et al., 2020).

#### From Chloroplasts

Chromoplasts synthesize and store carotenoids and are mainly found in petals and fruits, which are organs related to reproduction, but they can also occur in the leaves and roots. The transition from chloroplast to chromoplast starts from the breakdown of thylakoids and chlorophyll. This is followed by the increases of plastoglobuli size and the biosynthesis and accumulation of the carotenoids (Wrischer and Devide, 2002). Representative cases showing the irreversible process from chloroplast to chromoplast were found in tomato (*Solanum lycopersicum*) (Egea et al., 2011; Barsan et al., 2012; D’Andrea et al., 2014), red pepper (Jeong et al., 2020), Italian arum (Bonora et al., 2000), and lily (*Lilium longiflorum*) (Juneau et al., 2002). Interestingly, with single gene overexpression, such as the *AtPSY* overexpression line in Arabidopsis and the *Pantoea ananatis phytoene synthase* (*crtB*) overexpression by viral vector in tobacco were able to develop the chromoplast from leaf tissues (Maass et al., 2009; Majer et al., 2017). According to a recent report about the modifications of membrane structure in tomato chloroplasts, it was observed that the inner envelope membrane and thylakoid membranes disappeared during the transition to chromoplasts, and new factors (plastoglobules and crystal remnants, etc.) were generated through membrane fusion and vesicles budding (Wang et al., 2020).

### Development of Chloroplasts: De-Greening Phenotype

#### To Leucoplasts

One of the well-known examples for de-greening to leucoplast is Arabidopsis flower development. Young petals that have just bloomed contain green chloroplasts throughout their structures, but as the petals expand and develop, they lose chlorophyll and these regenerate into white bodies. During this process, plastids break down chlorophyll, and carotenoids are not synthesized (Pyke and Page, 1998; Irish, 2008).

#### To Gerontoplasts

Gerontoplasts are known to occur in both photosynthetic and non-photosynthetic organs as they appear with aging. In general, as chloroplasts age, although their outer shell remains intact, plastoglobuli are formed along with lipophilic substances, and extensive structural changes of the thylakoid membrane occur. In Arabidopsis, observations of gerontoplasts found that they had a degenerated outer shell and thylakoid membranes of chloroplast, enlarged plastoglobuli and grana, and these are gradually increased as they aged (Evans et al., 2010). Another study of the structural characteristics of gerontoplasts during *Jatropha curcas* seed development, found that the inner membrane system (thylakoid membrane and plastid outer envelope membrane) decomposed, and then there was gradual decomposition of the substrate and plastoglobuli (Shah et al., 2016).

## Regulators of Plastid Transitions

**FIGURE 2:**
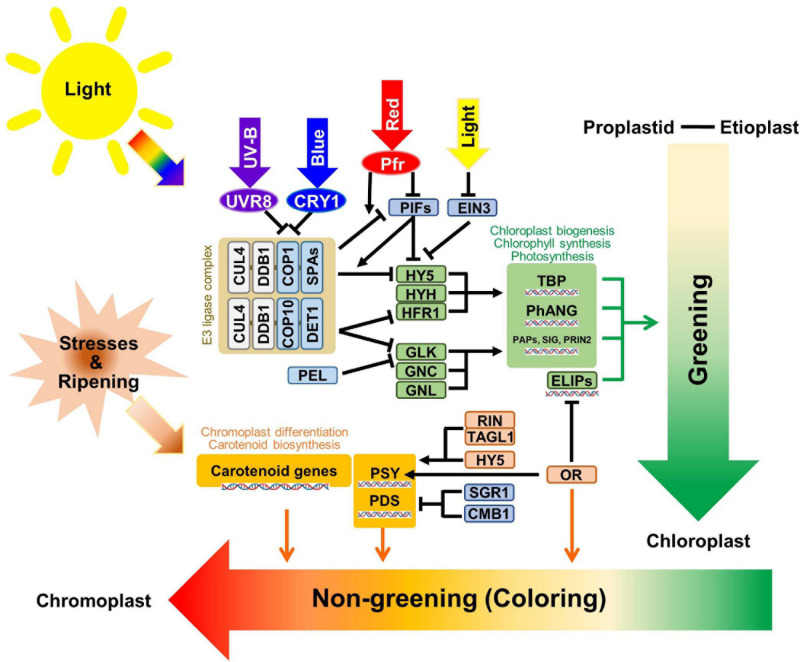
Schematic diagram of the plastid transition regulations. The regulatory mechanisms of greening and non-greening plastid interconversions are briefly summarized with core transcriptional and post-translational regulators. The light signals from different wavelengths are indicated with thick arrows with representative colors. The photoreceptors are shown with circles, while regulatory genes are shown within rectangular boxes. E3 ligase complexes, chloroplast biogenesis related enzymes, and chromoplast biogenesis related enzymes were categorized with white brown, green, and orange-colored boxes, respectively. DNA helix symbols represent the transcriptional regulation of genes. Green lines indicate the direct effects of “Greening” while red lines indicate the direct effects of “Non-greening”.

Recently, research on the regulatory pathways for plastids has largely expanded with many astonishing findings. So, due to considering the diversity and complexity of the plastid, most of the reviews for plastid related signals focused on specific aspects, such as plastid differentiation (Liebers et al., 2017; Knudsen et al., 2018; Sadali et al., 2019), light signals (López-Juez, 2007; Larkin and Ruckle, 2008; Larkin, 2014), redox control (Toyoshima et al., 2005), regulation and retrograde signals (Rodermel, 2001; Nott et al., 2006; Pogson et al., 2008; Kleine et al., 2009; Chi et al., 2013; Singh et al., 2015; Sun et al., 2018; Sun and Li, 2020), evolution (Reyes-Prieto et al., 2007; Archibald, 2009; Wicke et al., 2011; Keeling, 2013), and hormonal regulation (Al-Babili and Bouwmeester, 2015; Liu et al., 2017). In this review, we focused on the post-transcriptional regulation and newly found regulators for plastid transitions (Figure 2).

### Chloroplast Development

Light is the primary signal for chloroplast development. Different ranges in wavelength are perceived by different photoreceptors, for example, UV-B is identified by *UV RESISTANCE LOCUS 8* (*UVR8*), UV-A and blue light are identified by *cryptochromes* (*CRYs*) and phototropins, red light and far-red light are identified by *phytochrome-red* (*Pr*) and *phytochrome-far-red* (*Pfr*), respectively, as the interconvertible form of phytochromes (Paik and Huq, 2019). To take advantage of rapid response, the plants use post-transcriptional regulation by transferring the signal to E3 ligase mediated protein degradation pathways. In *CULLIN4* (*CUL4*) type E3 ligase complex, CUL4 and UV−DAMAGED DNA BINDING PROTEIN1 (DDB1) consist of fundamental structures while CONSTITUTIVE PHOTOMORPHOGENICs (COPs), SUPRESSOR OF phyAs (SPAs), and DE-ETIOLATED 1 (DET1) have target-specificity functions (Lau and Deng, 2012). The COP1-SPA1 complex is a core regulator for light perception by the UVR8, CRY1 receptors and phytochrome responses (Wang et al., 2001; Saijo et al., 2003; Huang et al., 2013; Martínez et al., 2018). When UVR8 and CRY1 are activated by light, they inactivate COP1 and exported COP1 protein to the cytosolic region from the nucleus and, consequently, block the ubiquitination of ELONGATED HYPOCOTYL 5 (HY5) protein (Lau and Deng, 2012). The COP10-DET1 complex was also reported to be involved in the ubiquitination of Long Hypocotyl in Far-Red 1 (HFR1) (Yang et al., 2005) and GOLDEN2-LIKE 1 (GLK1) (Tang et al., 2016). *HY5*, *HYH*, and *HFR1* were reported as positive transcriptional regulators used to assemble light signals for hypocotyl elongation and early light responses. GLKs also binds to the 32–88 aa region of DET1 and the protein stability of GLKs are increased by blocking ubiquitination mediated protein degradation (Tang et al., 2016). *GLKs* are core positive regulators for chloroplast development based on their mutant phenotypes, while the phenotype of *glk1/glk2* double KO still had chloroplasts which indicates the possibility of another regulator (Fitter et al., 2002; Wang et al., 2013). Two genes were reported for similar but minor phenotypes when compare to *GLKs*, called *GATA, NITRATE-INDUCIBLE, CARBON-METABOLISM INVOLVED* (*GNC*), and *GNC-LIKE* (*GNL*) (Richter et al., 2010). When the lights on, together with the release of the E3 ligase degradation pathway, there was transcriptional inhibition of *HY5*, *HYH*, and *HRF1* by PHYTOCHROME INTERACTING FACTORS (PIFs), and ethylene-insensitive 3 (EIN3) was released by the degradation of the PIFs and EIN3 (Zhong et al., 2009). This also triggered the transcriptional regulation of core positive regulators to give synergetic effects. *Pseudo-Etiolation in Light/DEEP GREEN PANICLE1* (*AtPEL1*) was primarily reported in the Arabidopsis Full-length cDNA Over-eXpressing gene hunting system (FOX hunting system) for the chlorophyll repression gene (Ichikawa et al., 2006; Llorente et al., 2017) and, interestingly, with the same FOX hunting system, the ectopic overexpression of *OsGLK1* convert proplastid into chloroplast in rice callus (Nakamura et al., 2009). The further analysis showed a binding affinity of OsPEL1 with OsGLK1 and OsGLK2 in rice. Although the regulatory model was not well-established and the function of PEL in rice was restricted to the panicle, AtPEL1 represses the activation activity of OsGLK1 (Zhang et al., 2020). With the assembly of positive regulators, the core enzymatic proteins for chloroplast biogenesis, the tetrapyrrole biosynthesis pathway, Photosynthesis Associated Nuclear Genes and PEP-associated proteins (PAPs), SIGMA factors (SIG), PRIN2 were elevated (Kindgren et al., 2012; Kobayashi and Masuda, 2016; Hernández-Verdeja et al., 2020).

### Chromoplast Development

Several regulators play essential roles in chromoplast transition, but they are not as well-established as in chloroplasts. During fruit ripening in tomato *(Solanum lycopersicum*), the MADS-box transcription factor *RIPENING INHIBITOR* (*RIN*) has been reported as core positive regulator that activates rate-limiting enzymes in carotenoid pathways (Martel et al., 2011). TOMATO AGAMOUS LIKE1 (TAGL1) forms a complex with RIN and lycopene in tomato fruits was fortified by *TAGL1* overexpression (Itkin et al., 2009; Lü et al., 2018). A non-functional mutant of the NAC transcription factor called *NON-RIPENING* (*NOR*) was reported for a similar phenotype of the *rin* mutant by retardation of the plastid transition (Giovannoni, 2004, 2007). A light-induced bZIP transcription factor *HY5* directly binds to a promoter for carotenoid biosynthesis rate-limiting enzymes, *PSY* and phytoene desaturase (*PDS)*, and activates their transcription (Toledo-Ortiz et al., 2014). Another positive regulator, *OR*, was responsible for the generation of chromoplasts in floral organs of cauliflower (*Brassica oleracea* var. botrytis) (Li et al., 2003; Lu et al., 2006). OR was also reported to have holdase chaperone activity for *PSY*, as it enhanced the PSY protein stability and increased its enzymatic activity (Welsch et al., 2018). In Arabidopsis, further analysis found that the OR binds with a bHLH transcription factor TEOSINTE BRANCHED, CYCLOIDEA AND PCF (TCP14) to increase the stability and transcription level of *EARLY LIGHT-INDUCIBLE PROTEINS* (*ELIP1* and *ELIP2*), which are chlorophyll binding proteins for chloroplast development (Sun et al., 2019). And diverse mutant analysis with different species indicates the essential roles of OR gene in chromoplast development (Kim et al., 2018; Welsch et al., 2020). Meanwhile, as repressive regulators, STAY-GREEN 1 (SlSGR1) in tomato directly interacted with SlPSY1 and could inhibit its protein levels. Furthermore, the repression of *SlSGR1* in transgenic tomato fruits resulted in the elevation of *SlPSY1* mRNA accumulations and the acceleration of chromoplast interconversion times (Luo et al., 2013). One of the MADS-box proteins called *SlCMB1*, which belongs to the same SEP sub-clade with *RIN*, was also reported to have an essential role in chromoplast development in tomato. The *SlCMB1*-RNAi fruits have decreased *PSY1* and *PDS* expression, and decreased ethylene production and related signal pathway genes (Zhang et al., 2018). Furthermore, the exogenous induction of rate-limiting carotenoid biosynthesizing enzymes able to trigger the interconversion of plastids to chromoplasts (Ha et al., 2019; Llorente et al., 2020). Diverse environmental stresses and developmental signals including ripening could also trigger chromoplast development through the induction of carotenoid biosynthesis (Bouvier et al., 1998; Sadali et al., 2019).

## Conclusion and Perspective

From the overview of diverse representative, sophisticate adjustment of plastid interconversion revealed as essential element for multiple agronomic traits. Not only the photosynthesis related yield potential, many plant-derived biproducts including starch, lipid, protein and secondary metabolites have been determined by development and interconversion of plastid. Although, under the demands from the plants are continuously increased with limited environmental condition, this review suggest the study of plastid can be the breakthrough solution and supports the plastid research by representative scientific reports of plastid interconversion types and core regulators for molecular modification candidates. Finally, the candidates from the well summarized molecular pathway for plastid interconversion can be applied as target gene for improving multiple agronomic traits which are related to plastid interconversion.

## Author Contributions

S-HH designed the concept and the organization of the manuscript. HC and TY wrote and edited the manuscript. All authors have read and approved the final manuscript.

## Conflict of Interest

The authors declare that the research was conducted in the absence of any commercial or financial relationships that could be construed as a potential conflict of interest.
